# Involvement of YAP-1, the Homolog of Yes-Associated Protein, in the Wnt-Mediated Neuronal Polarization in *Caenorhabditis elegans*

**DOI:** 10.1534/g3.118.200325

**Published:** 2018-05-31

**Authors:** Hanee Lee, Junsu Kang, Junho Lee

**Affiliations:** Department of Biological Sciences, Institute of Molecular Biology and Genetics, Seoul National University, Gwanak-ro 1, Gwanak-gu, Seoul, Korea 08826

**Keywords:** *C. elegans*, neuronal asymmetry, development, YAP-1, the Wnt pathway

## Abstract

Guidance molecules, receptors, and downstream signaling pathways involved in the asymmetric neuronal cell migration and process outgrowth have been identified from genetic studies using model organisms, most of which are evolutionarily conserved. In the nematode *Caenorhabditis elegans*, the roles of Wnt ligands and their receptors in the polarization of specific sets of neurons along the anterior-posterior (A-P) body axis have been well elucidated, but their downstream effectors are relatively unknown. Here, we report *yap-1*, encoding an evolutionarily conserved transcriptional co-activator, as a novel player in the Wnt-mediated asymmetric development of specific neurons in *C. elegans*. We found that the loss of *yap-1* activity failed to restrict the dendritic extension of ALM neurons to the anterior orientation, which is similar to the phenotype caused by defective *cwn-1* and *cwn-2* Wnt gene activities. Cell-specific rescue experiments showed that *yap-1* acts in the cell autonomous manner to polarize ALM dendrites. We also found that subcellular localization of YAP-1 was spatio-temporally regulated. The loss of *yap-1* in Wnt-deficient mutants did not increase the severity of the ALM polarity defect of the mutants. Wnt-deficient animals displayed abnormal subcellular localization of YAP-1 in touch receptor neurons, suggesting that *yap-1* may act downstream of the *cwn-1/cwn-2* Wnt ligands for the ALM polarization process. Together, we have identified a new role for YAP-1 in neuronal development and our works will contribute to further understanding of intracellular events in neuronal polarization during animal development.

Establishment of structural and functional polarity is an essential step in neuronal development. For several decades, many evolutionarily conserved guidance molecules and downstream signaling directing these processes have been uncovered from model organisms. Due to the simplicity and invariance between individuals, the *C. elegans* nervous system has been widely used as a model to identify neuronal guidance cues, which have been revealed to be evolutionarily conserved from worms to vertebrates. For example, the six touch receptor neurons (TRNs), consisting of 2 ALM, AVM, 2 PLM and PVM neurons, have been extensively studied to elucidate the mechanism of neuronal asymmetry, because they display a typical polarity along the anterior-posterior (A-P) and the dorsal-ventral (D-V) body axis of the worm (Chalfie *et al.* 1985). So far, many polarity determinants of TRNs have been identified. UNC-6/Netrin and its receptor UNC-40/Frazzled/DCC were found to direct the ventral movement of AVM and PVM cell bodies (Hedgecock *et al.* 1990; Ishii *et al.* 1992) and their functions in the ventral guidance are conserved in developing nervous systems of fly and mammals (Harris *et al.* 1996; Hiramoto *et al.* 2000; Serafini *et al.* 1994; Kennedy *et al.* 2006; Mitchell *et al.* 1996). In the case of neuronal orientation along the A-P axis, the roles of the Wnt pathway have been well established. Wnt proteins, conserved in all metazoan, are secreted glycoproteins that transmit intracellular signals through their receptors, Frizzled, Ryk or Ror. In early embryos, Wnt proteins form gradients and mediate many polarized developmental aspects including not only the asymmetric development of neurons but also early patterning of embryos (Hilliard and Bargmann 2006; Pan *et al.* 2006; Petersen and Reddien 2009; Prasad and Clark 2006; Silhankova and Korswagen 2007). Although the redundant roles of Wnt ligands and their receptors in the polarization of ALM and PLM along the anterior-posterior (A-P) body axis have been well elucidated, their downstream effectors are relatively unknown.

The Hpo signaling pathway is an evolutionarily conserved pathway that regulates many aspects of development. Upstream signals activate the kinase cascade of the pathway and activated LATS kinase phosphorylates and inhibits nuclear localization of YAP or TAZ, the transcriptional co-activator of TEAD transcription factor. In the nucleus, YAP and TEAD regulate transcriptions of target genes such as *cycE* and *bantam* which are mainly acting cell proliferative roles (Hu *et al.* 2004; Harvey *et al.* 2003; Wei *et al.* 2007). The Hpo pathway is well known for regulating tissue size homeostasis via controlling cell proliferation and apoptosis as shown in fly and mammals (Pan 2010). It also functions in the nervous system, where it is also involved in regulating cell proliferation as it usually functions in the early development of neurons including neuronal progenitor cell proliferation. In the developing vertebrate neural tube, the loss of LATS activity leads to the expansion of neuronal progenitor pools in the YAP and TEAD activity-dependent manner (Fernandez-L *et al.* 2009; Cao *et al.* 2008). On the contrary, some pieces of evidence show that Hpo and Wts act in dendrite tiling and the maintenance of sensory neurons in fly (Emoto *et al.* 2006; Parrish *et al.* 2007). In addition, *sax-1*, the worm homolog of NDR kinase, which is a conserved subclass of the AGC group kinase together with LATS (Pearce *et al.* 2010), is involved in dendrite termination of TRNs (Gallegos and Bargmann 2004) independently of their functions in cell proliferation. In *C. elegans*, several components of the Hpo pathway and their genetic interactions are conserved (Kang *et al.* 2009; Cai *et al.* 2009; Iwasa *et al.* 2013); *wts-1*, *yap-1*, and *egl-44* are the worm homolog of LATS, YAP and TEAD, respectively (Iwasa *et al.* 2013). Interestingly, our previous study showed that WTS-1 is not involved in cell proliferation or apoptosis but in apical membrane polarity maintenance (Kang *et al.* 2009). Considering that *orb6*, a LATS homolog in yeast, is also required to maintain cell polarity (Verde *et al.* 1998), roles of the Hpo pathway are not limited to proliferation but extended to the cellular polarity maintenance, which may reflect evolutionarily ancient roles of the Hpo pathway. Together, despite the possible roles of the Hpo pathway in the asymmetric differentiation of neurons, any definitive proof has not been provided yet.

Here, we report that YAP-1, the worm homolog of YAP, is required for neuronal polarization along the A-P axis in *C. elegans*. *yap-1* mutant animals display defects in the asymmetric extension of ALM neurites and our genetic works prove that *yap-1*, possibly acting downstream of specific Wnt ligand genes, functions in neuronal asymmetric development in the cell- autonomous manner. Our works define a new physiological role of YAP-1 in *C. elegans*, which may imply the evolutionary significance of the involvement of YAP-1 in cellular polarity in other animals including humans.

## Materials and Methods

### The Nematode Strains

Worms were grown at 20° and handled with the standard methods (Brenner 1974). Following strains were used. N2 Bristol, SK4005 *zdIs5* [*mec-4*p::GFP, *lin-15(+)*], *yap-1(tm1416)*, *yap-1(ys37)*, *yap-1(ys38)*, RB763 *cwn-1(ok546)*. VC636 *cwn-2(ok895)*. EW72 *cwn-1(ok546)*; *cwn-2(ok895)*. To visualize touch receptor neurons in various mutants, we transferred *zdIs5* [*mec-4*p::GFP, *lin-15(+)*] into each genetic background by mating.

### Molecular biology and Transgenic lines

To rescue YAP-1 activity, a full genomic coding region of YAP-1 with its own 4 kb promoter was cloned into the GFP-containing pPD114.108 using a standard subcloning method. To express YAP-1 TRNs specifically, GFP fused YAP-1 was cloned into pCFJ150 under *mec-4* promoter using the Multisite Gateway system (Invitrogen, Inc.). To obtain transgenic lines, each plasmid was injected into worms at 50 ng/μl with 50 ng/μl of pRF4 as a transgenic marker. To monitor the subcellular localization of YAP-1 in TRNs of wild type and worms lacking Wnt activities, *mec-4*p::GFP::YAP-1 was injected into the wild type and transgenes were transferred to each mutant background by mating.

### Analysis of ALM morphology and subcellular localization of YAP-1

Worms were mounted on 3% agar pads and immobilized using 2.5 mM levamisole. To score ALM morphology, about 20 worms at L1 or L4 stage were observed in one experiment and experiments were repeated for 3 times. The phenotype was categorized as either one of the following: with no posterior process, with short posterior process (<2 ALM cell diameter), with intermediate posterior process (<5 ALM cell diameter), or with long posterior process (longer that 5 ALM cell diameter). Among these, we defined as defective ALM with intermediate or long posterior process. To observe YAP-1 subcellular localization in ALM, about 20 gravid adult worms were transferred to an NGM plate. After 2 hr, worms were removed and remained eggs and hatched larvae after several hours were used for observation. Experiments were repeated for 3-4 times. Fluorescence images were acquired using the confocal microscope (ZEISS LSM700, Carl Zeiss, Inc.) and ZEN software (Carl Zeiss, Inc.).

### Immunofluorescent staining

Immunofluorescent staining of YAP-1 was done by the freeze-crack method, as previously described(Bowerman *et al.* 1993). Bleached embryos and newly hatched larvae of wild type or *cwn-1*; *cwn-2* mutant expressing *mec-4*p::GFP::YAP-1 were fixed by 5% PFA. Touch neuronal YAP-1 was detected by TRITC conjugated GFP antibody (sc-9996, 1:100) and DNA was stained with 4e, 6-diamidino-2-phenylindole (DAPI). Images were obtained using a confocal microscope (ZEISS LSM700, Carl Zeiss, Inc).

### RNA preparation, cDNA synthesis and quantitative real time PCR analysis

Embryonic total RNA was isolated from bleached eggs of each strain with TRI reagent (Molecular Research Center, Inc.) by standard freeze-thaw method. cDNA was synthesized with TOPscript reverse transcriptase(Enzynomics, Inc.) using oligo(dT) primers and used as PCR templates. Quantitative real time PCR was performed using BIO-RAD iQ SYBR Green supermix in BIO-RAD CFX connect as described in manufacturer’s manual. Primers used in PCR were generated by primer3 (http://bioinfo.ut.ee/primer3-0.4.0/primer3/). The expression level of *act-1/3* was used for normalization. Three biological replicates were used for analysis.

### Data availability

Strains and plasmids are available upon request. The authors state that all data necessary for confirming the conclusions presented in the article are represented fully within the article.

## Results and Discussion

### *yap-1* is required for establishing the polarized neurites of ALM neurons

**Figure 1:**
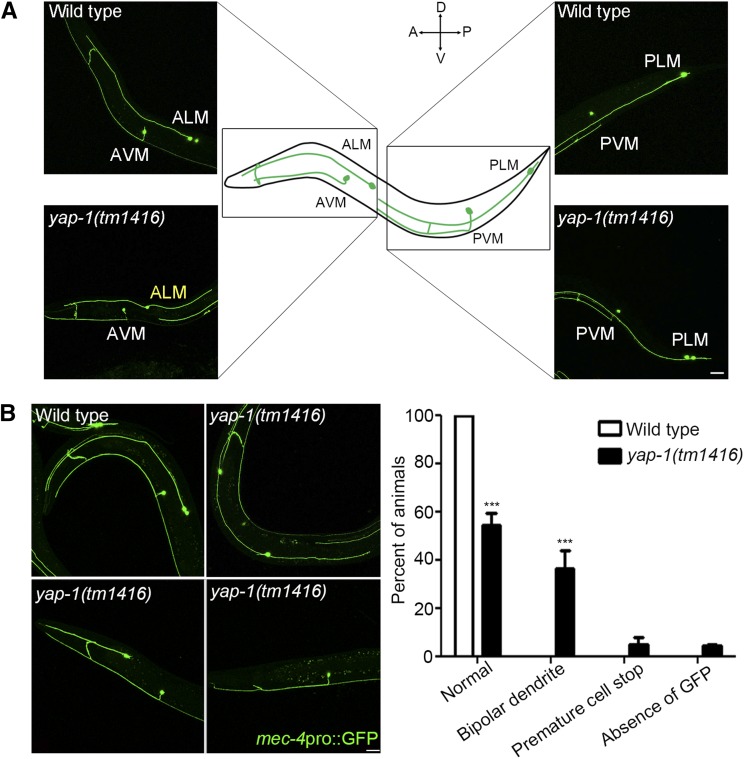
Loss of YAP-1 abrogates ALM (A) Morphology of Touch Receptor Neurons (Green) in wild type and *yap-1* mutant animals. TRNs are labeled with *zdIs5* [*mec-4*p::GFP]. (B) ALM neuron of wild type and *yap-1(tm1416)*. *yap-1(tm1416)* shows various defects in ALM including polarity defect, premature cell stop and the loss of GFP expression driven by *mec-4* promoter in ALM. ALM were observed at L4 stage. Scale bar: 20 μm. In the graph, shown are average percentages of neurons ± SD ***, different from wild type, *P* < 0.001. Statistical significances were determined by two-way ANOVA, followed by Bonferroni comparison.

In order to examine the role of *yap-1* in cell polarity in neurons, which may be reflected in neuronal asymmetry, we decided to examine the neuronal morphology of touch receptor neurons (TRNs) in *yap-1* mutant animals. We chose TRNs because these neurons are morphologically asymmetric and visually easy to observe. While the morphology of the other TRNs was not significantly different from those of wild type, ALM morphology was defective in *yap-1(tm1416)* mutant animals (Figure 1A, B). During normal development, ALM cell bodies posteriorly migrate to the middle region of the body, and anteriorly extend their neurites to the nerve ring. Thus, matured ALM neurons are polarized along the A-P body axis (Figure 1A). Worms that have a putative null mutation in the *yap-1* showed various morphological defects in ALM neurons at their L4 stage. They often extended dendritic processes not only to the anterior region but also to the posterior region (36.29%). They also displayed premature stop of cell body migration (4.79%) and frequently lost GFP expression driven by *mec-4* promoter only in ALM (4.43%) (Figure 1B). The *mec-4* encodes a probable sodium channel subunit and is specifically expressed in six TRNs. Thus, it is possible that the specific loss of *mec-4* expression is possibly due to the changes in cell fates of ALM neurons.

**Figure 2:**
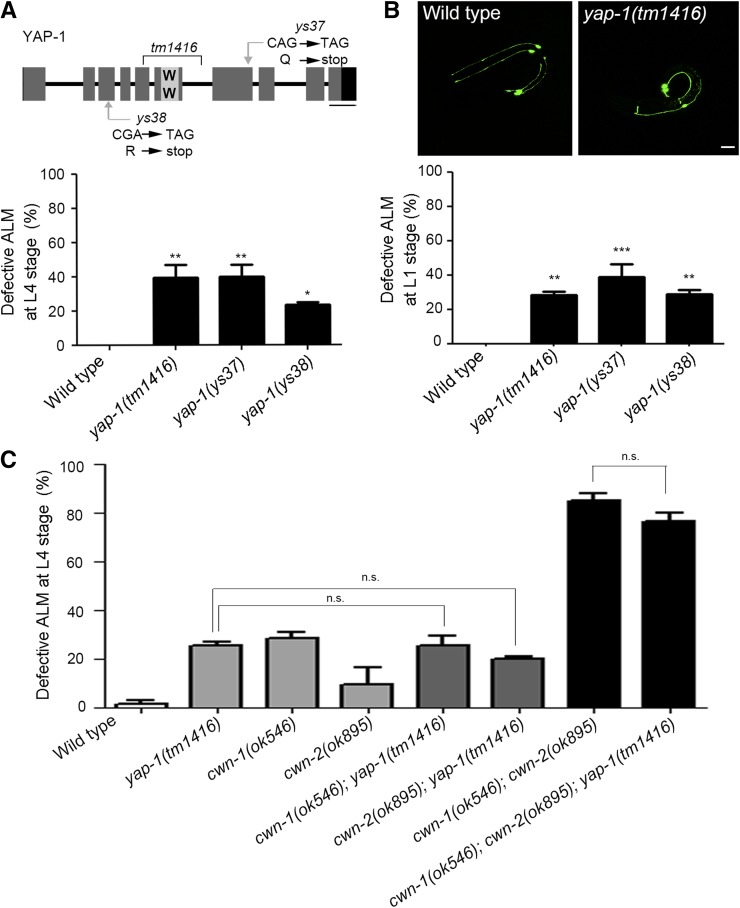
*yap-1* may act in the Wnt- mediated asymmetric development of ALM (A) The frequency of defective polarity of ALM in wild type, *yap-1(tm1416)*, *yap-1(ys37)* and *yap-1(ys38)* at L4 stage. Mutation sites of each allele are annotated in the upper YAP-1 gene structure diagram. Scale bar: 200 bp (B) The frequency of ALM polarity defects of various alleles of *yap-1* at early L1 stage. *yap-1* show significant loss of ALM asymmetry from the very early L1 stage. Upper panels show representative images and quantified results were followed. Scale bar: 10 μm. (C) Quantification of the defective polarity of ALM in various mutants with *yap-1* and the Wnt ligands, *cwn-1 and cwn-2*. ALM neurons were observed at L4 stage. (A-C) The frequency of defective polarity of ALM was calculated as the ratio of the number of bipolarized ALM over the number of bipolarized and unipolarized ALMs. We excluded ALM in which polarity was reversed or GFP driven by the *mec-4* promoter was lost from total number. Statistical differences were determined by one-way ANOVA, followed by Turkey’s multiple comparison test. Asterisks indicate differences from wild type. ***, *P* < 0.001, **, *P* < 0.01, *, *P* < 0.05, n.s., not significant.

**Figure 3:**
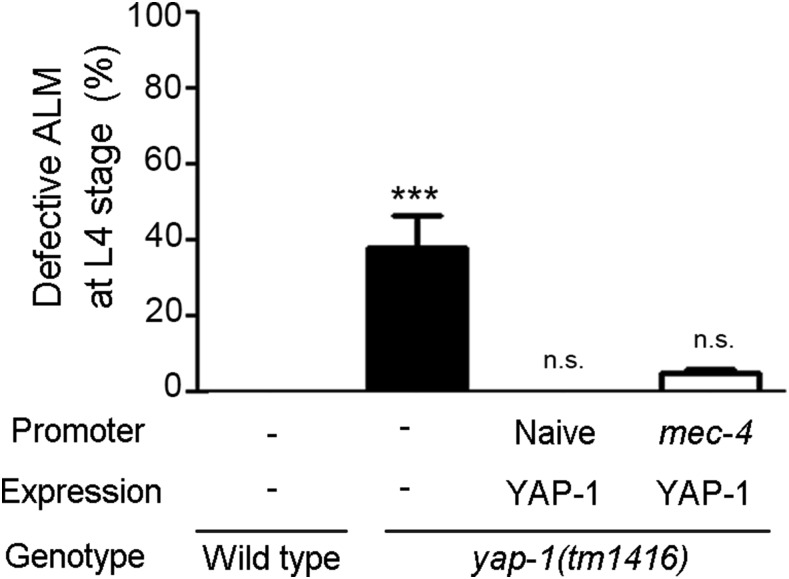
YAP-1 acts cell autonomously to polarize ALM Results of cell type-specific rescue of YAP-1 activity. In *yap-1(tm1416)*, YAP-1 activity was rescued by own promoter (4 kb) or *mec-4* promoter which expresses specifically in TRNs. ALM neurons were scored at L4 stages. Statistical differences were determined by Dunnett’s multiple comparison test. Asterisks indicate differences from wild type. ***, *P* < 0.001, n.s., not significant.

Among the phenotypes observed, the defective polarity of ALM neurons was the most prominent phenotype of mutants, therefore we next focused on the roles of YAP-1 in the asymmetric neurite extension of ALM neurons. We examined two more *yap-1* mutations to confirm that the phenotype observed is due to the *yap-1* mutations, not by other background mutations. The *yap-1*(*ys38*) mutation has a point mutation that results in a premature stop in the coding region of YAP-1 and the *yap-1*(*ys37*) mutation produces a truncated YAP-1 protein displaying a dominant negative effect (Figure 2A). Both *yap-1*(*ys37*) and *yap-1*(*ys38*) mutant animals showed the defective polarity phenotype at their L4 stage with similar penetrance to that of *yap-1*(*tm1416*) (Figure 2A). Among Six TRNs, ALM and PLM, the posterior homolog of ALM, are known to terminate development during embryogenesis. To define whether *yap-1* is required for development or maintenance of asymmetry of ALM neurons, we observed ALM neurons in wild type and the various mutant alleles of *yap-1* at the very early L1 stage. As reported, in just-hatched wild type worms, ALM and PLM neurons showed apparent polarity along the A-P axis, whereas AVM and PVM neurons that differentiate in the post-mitotic period had not been generated (Figure 2B). We found that just-hatched *yap-1* mutant larvae showed a similar frequency of defective polarity of ALM neurons to that of L4 *yap-1* mutants (Figure 2B). In addition, the extrachromosomal expression of YAP-1 under the control of its own promoter (4 kb) was sufficient to fully restore anterior extension of ALM dendrite in *yap-1* mutant (Figure 3). These results suggest that YAP-1 is required to establish ALM polarity, not to maintain it.

### *yap-1* acts in same genetic pathway With specific Wnt ligand genes

In the past decades, many polarity determinants of *C. elegans* neurons were identified. Slit/Netrin signaling mainly regulates neuronal development along Dorsal-Ventral Axis and the highly conserved Wnt pathway has been known to orient cell migration and neurite outgrowth along the A-P axis (Killeen and Sybingco 2008; Silhankova and Korswagen 2007). Wnt redundantly acts to establish the polarity of touch receptor neurons including ALM, thus Wnt lacking mutants exhibit polarity defects of ALM or PLM from newly hatched larva stage (Prasad and Clark 2006; Hilliard and Bargmann 2006). Whereas, a mutation of *mec-7*, β-tubulin, leads failures in the maintenance of neuronal polarity. ALM of a *mec-7* mutant displays normal anteriorly oriented process at birth. It gradually extends the ectopic posterior process and lose its axon-dendrite polarity (Kirszenblat *et al.* 2013).

To define YAP-1 functions in the establishment of ALM polarity, we examined whether YAP-1 acts in the same genetic pathway with Wnt, the most well identified anterior guidance cues of ALM neurons. The *C. elegans* genome encodes 5 Wnt ligands *(mom-2*, *lin-44*, *cwn-1*, *cwn-2* and *egl-20)*, 4 Frizzled/Fz receptors (*mig-1*, *lin-17*, *cfz-2* and *mom-5*), 1 Ryk (*lin-18*) and 1 Ror (*cam-1*). In ALM polarization, three of the Wnt ligands (*cwn-1*, *cwn-2* and *egl-20*) and their receptors, *cam-1* and *mom-5* act redundantly to direct neuronal asymmetry (Hilliard and Bargmann 2006; Pan *et al.* 2006; Prasad and Clark 2006; Chien *et al.* 2015). Because canonical Wnt pathway components including *bar-1*/β-catenin and *pop-1*/Tcf were not necessary for ALM polarization, it has been suggested that a non-canonical Wnt pathway, which is independent of β-catenin, mediates ALM polarization (Chien *et al.* 2015). While worms lacking both *cwn-1* and *egl-20* display reversely polarized ALM neurons in which ALM neurons extend processes only toward in the posterior direction, double mutants lacking both *cwn-1* and *cwn-2* have symmetrically extended ALM neurites(Prasad and Clark 2006). The latter phenotype was similarly shown in *yap-1* mutant animals. Thus, we generated and examined the combinatorial mutants between *yap-1* and Wnt ligand genes *cwn-1* and *cwn-2* to elucidate the relationship among these genes. We found that the introduction of *yap-1* null mutation into *cwn-1* or *cwn-2* single mutants or to *cwn-1;* *cwn-2* double mutant animals did not cause further increase in the bipolar defect of ALM (Figure 2C), suggesting that YAP-1 may act in the same genetic pathway with the Wnt genes for the asymmetric development of ALM neurons. In addition, loss of *yap-1* did not enhance reversely oriented phonotype of *cwn-1;* *cwn-2*. 12.90±3.59% of *cwn-1;* *cwn-2* showed only posteriorly oriented ALM processes, whereas 10.47± 1.67% of *cwn-1;* *cwn-2;* *yap-1* did. As reported, although ALM and other TRNs, PLM specifically, display morphological similarity along body axis, they used different repertoire of Wnt ligands, *lin-44* and corresponding receptors, *lin-17*. It is conceivable that YAP-1 acts, in same genetic pathway with CWN-1 and CWN-2, but probably not with EGL-20, to specifically polarize ALM neurons anteriorly and does not mediate development of other TRNs. However, the penetrance of the ALM phenotype of *yap-1* single mutant is lower than that of *cwn-1;* *cwn-2*, suggest that it is possible that other factors we do not yet know act in Wnt-mediated ALM polarity establishment in parallel with *yap-1*.

### *yap-1* acts cell autonomously to polarize ALM dendrites

Then, to determine where YAP-1 acts to polarize ALM neurites, we made transgenic worms in which YAP-1 activity was rescued only in touch receptor neurons. TRNs-specific expression of YAP-1 by the *mec-4* promoter was sufficient to rescue the polarity defect of ALM neurons in *yap-1* mutant animals (Figure 3). It suggest that YAP-1 activity is required for the asymmetric development of ALM neurons in a cell autonomous manner.

**Figure 4:**
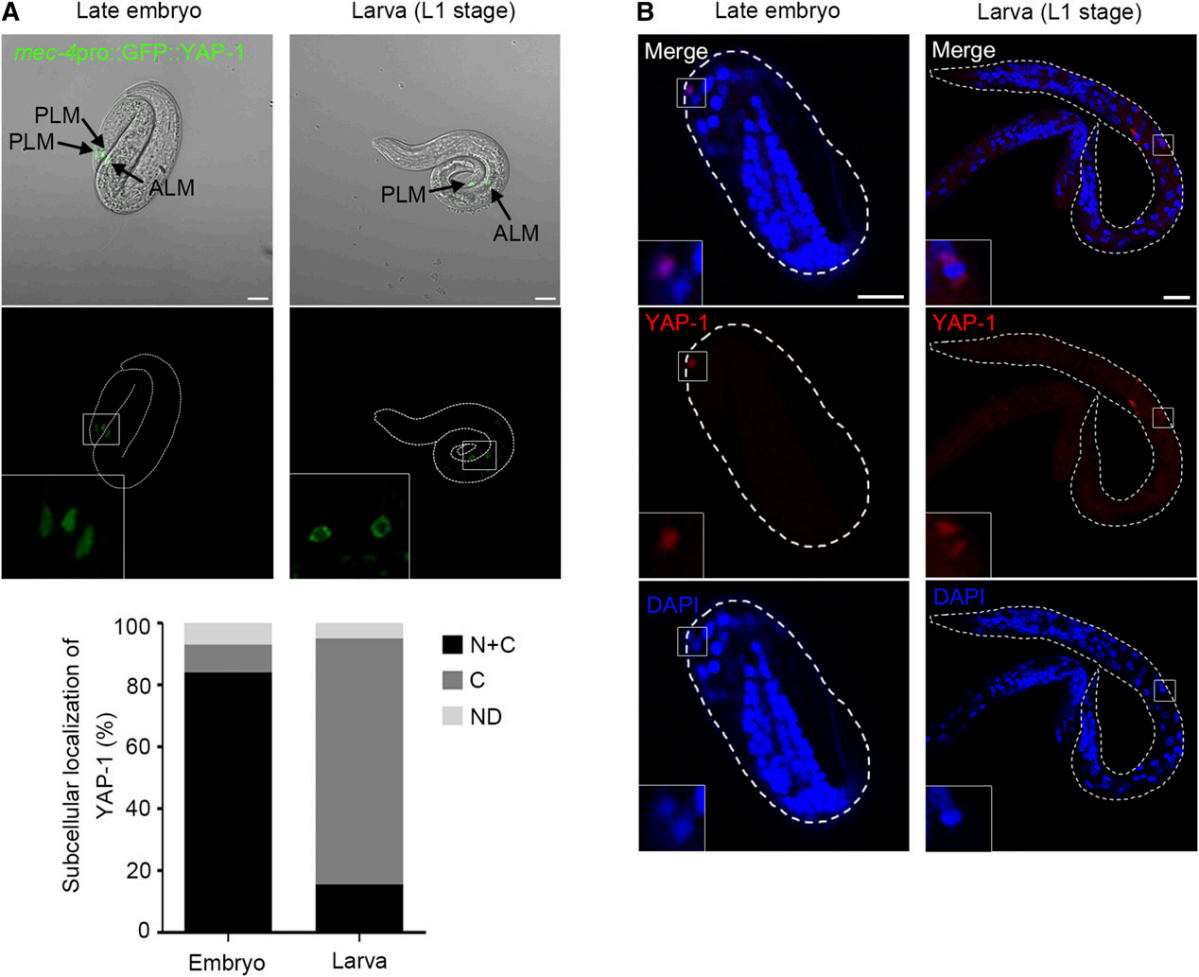
Spatiotemporal regulation of YAP-1 in ALM (A) Subcellular localization of YAP-1 in embryonic and larval TRNs. In late embryos, YAP-1 localized both in nucleus and cytoplasm of TRNs. In contrast, YAP-1 exclusively localized in the cytoplasm of TRNs in nearly all L1 larvae. TRNs specific expression of GFP-tagged YAP-1 was driven by *mec-4* promoter. Scale bars: 10 μm. The lower graph shows quantified results of subcellular localization of YAP-1 in TRNs. Totally, 112 eggs and 98 larvae were scored. (B) Immunofluorescent staining of YAP-1 in touch receptor neurons. GFP fused YAP-1 was detected by TRITC conjugated GFP antibody. YAP-1 (red) and 4′, 6-diamidino-2-phenylindole, DAPI (blue) for DNA. The left panels are wild type embryo and the right panels, L1 larvae. Scale bars: 10 μm.

As previously described, YAP-1 is a worm homolog of YAP that acts as a transcriptional co-activator whose subcellular localization is tightly regulated by the upstream Hpo pathway. In worms, endogenous YAP-1 is expressed in the various epithelial cells including intestine and hypodermis, and its nuclear localization is limited in the embryonic stage (Iwasa *et al.* 2013). To determine whether the regulatory mechanism of YAP-1 exists in ALM neurons, we examined subcellular localization of YAP-1 in touch receptor neurons. Although YAP-1 expression under the control of its own promoter was sufficient to rescue the mutant phenotype, naïve expression of YAP-1 in neurons was too faint for accurate observation. Thus, to monitor the subcellular localization of YAP-1 in ALM neurons, we introduced the GFP tagged-YAP-1 construct under the *mec-4* promoter, which was used to rescue YAP-1 activity in TRNs. We found that the subcellular localization of YAP-1 was also spatio-temporarily regulated in TRNs. GFP-fused YAP-1 under the control of the *mec-4* promoter was localized in the ALM nuclei only in embryos. After hatching, YAP-1 was sequestered in the cytoplasm in the almost animals we observed (Figure 4A). YAP-1 occasionally appeared to form cytoplasmic puncta in larvae (Figure 4A, right panels). To more closely examine the intracellular localization of YAP-1 in touch receptor neurons, we performed immunofluorescent staining of touch neuronal YAP-1. Worms expressing GFP-fused YAP-1 under the *mec-4* promoter were used for the analyses and YAP-1 was detected by the anti GFP antibody. We found that nuclear YAP-1 was detectable only in embryos (Figure 4B). Although loss of *yap-1* did not lead to any detectable defect in PLM development, this spatiotemporal regulation of YAP-1 also existed in PLM (Figure 4A). This spatiotemporal regulation of YAP-1 in TRNs is consistent with that in the intestine and the hypodermis (Iwasa *et al.* 2013). Considering that ALM neurons complete their development during embryogenesis, these data collectively suggest that YAP-1 enters the nucleus of ALM neurons and functions in the asymmetric development of ALM neurons in the cell autonomous manner, possibly regulating transcriptional regulation of target genes.

### The Wnt pathway may regulate *yap-1* at the level of transcription and protein localization

**Figure 5:**
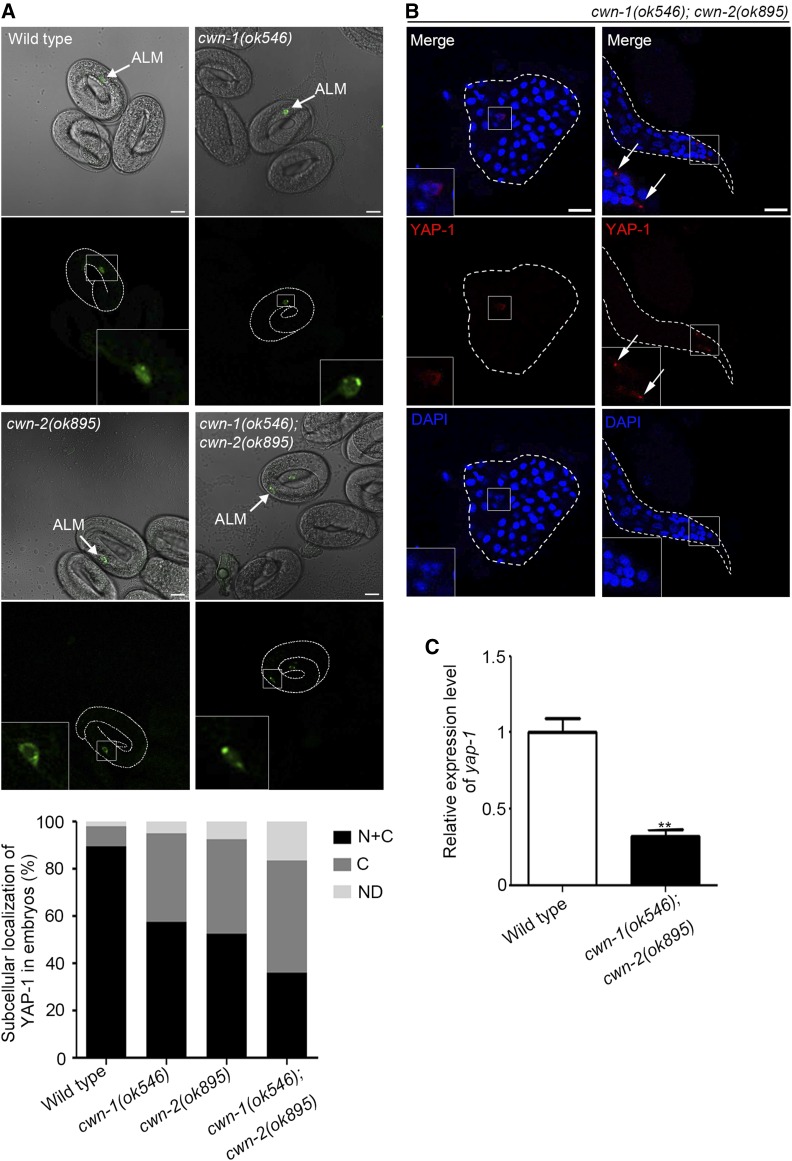
The Wnt is involved in the regulation of YAP-1 **(**A) Subcellular localization of YAP-1 in embryonic ALM in wild type and mutant lacking the Wnt. Scale bars: 10 μm. The lower graph displays the quantified subcellular localization of YAP-1 in embryonic ALM of each strain. Total numbers of embryos were counted as follow: 83 wild type; 75 *cwn-1(ok546)*; 67 *cwn-2(ok895)*; 78 *cwn-1(ok546)*; *cwn-2(ok895)*. (B) Immunofluorescent staining of YAP-1 in touch receptor neurons of the Wnt lacking mutants, *cwn-1(ok546)*; *cwn-2(ok895)*. White arrows indicate cytoplasmic puncta of YAP-1. Scale bars: 10 μm. (C) Relative expression level of *yap-1* in wild type N2 and *cwn-1*; *cwn-2* mutant. Total RNAs were precipitated from embryos of each strain. Statistical significance was determined by unpaired student’s *t*-test. **, *P* < 0.01.

Recently, the crosstalk between Wnt signaling and Hpo signaling pathway has been intensively studied in the mammalian system and revealed to be highly complex depending on the context. In the mammalian intestine, YAP1 maintains the cell proliferation of crypt stem cells by activating Wnt pathway (Zhou *et al.* 2011; Camargo *et al.* 2007). On the contrary, several studies show that the cytoplasmic YAP1 and TAZ inactivates the canonical Wnt signaling pathway by inhibiting nuclear localization of β-catenin (Barry *et al.* 2013; Azzolin *et al.* 2014; Imajo *et al.* 2012). Furthermore, some evidence has shown that Wnt pathway acts as an upstream activator of YAP/TAZ via inhibiting LATS kinase or directly promoting YAP/TAZ transcription (Azzolin *et al.* 2012; Konsavage *et al.* 2012; Park *et al.* 2015). Considering that the Wnt ligands are secreted and transmit signals into target cells through their receptors and that YAP-1 acts cell autonomously in ALM, we hypothesized that YAP-1 may act downstream of the Wnt signaling in ALM to polarize neurite outgrowth. To figure out how Wnt ligands regulate *yap-1* activity in ALM, we observed the subcellular localization of YAP-1 in touch receptor neurons of Wnt-lacking worms. In Wnt-lacking larvae, touch neuronal YAP-1 was localized in the cytoplasm and sometimes it formed puncta, as in normal individuals (Figure 5B, right panels). However, in embryos of *cwn-1, cwn-2* single mutants or *cwn-1; cwn-2* double mutant, the ratios of nuclear localized YAP-1 were decreased in compared to that of wild type. Unlike in wild type embryos, cytoplasmic YAP-1 was often observed in Wnt-lacking embryos (Figure 5A, B). It indicates that the nuclear localization of YAP-1 in developing ALM requires the Wnt ligand genes. Unexpectedly, *cwn-1/cwn-2* activities were also needed for proper regulation of YAP-1 in PLM. In all embryos, subcellular localization of YAP-1 in ALM and PLM was nearly identical. It seems that although the Wnt-mediated spatiotemporal regulation of YAP-1 also works in PLM, YAP-1 activity is not essential for PLM polarization.

Another possible, but not mutually exclusive, mechanism is that the Wnt pathway regulates *yap-1* transcription. Consistent with this idea, the relative expression level of *yap-1* was significantly decreased in the *cwn-1; cwn-2* mutant embryos (Figure 5C). Since YAP-1 own promoter is notably expressed in hypodermis not in touch receptor neurons, more precise measurement such as smFISH will be needed to detect and quantify YAP-1 rare transcripts in ALM neuron of wild type and Wnt lacking mutant. Also, further studies are needed to determine whether absence of the Wnt directly reduce *yap-1* expression because β-catenin is required for the Wnt-mediated transcriptional regulation of YAP in other system. Investigation of involvement of other Hpo pathway components including *wts-1* in ALM polarization will be needed to elucidate molecular mechanism of YAP-1 regulation by the Wnt.

### Summary and perspectives

To summarize, our study reveals the novel role of YAP-1 in asymmetric neurite orientation of neurons in *C. elegans*. We found that YAP-1 acts in ALM polarization along A-P axis cell autonomously and also found that YAP-1 may function in the same genetic pathway with the Wnt ligands, the most known regulator of ALM polarization. And our observations suggest that the Wnt mediates spatiotemporal regulation of YAP-1 in developing ALM and imply the role of YAP-1 as a downstream effector of the Wnt mediated ALM polarization. Further study to clarify the molecular link between YAP-1 and the Wnt pathway will lead us to further understanding of intracellular events of polarized cells. And it would be interesting to figure out whether the function of YAP-1 and interaction between YAP and the Wnt pathway in neuronal asymmetric development is conserved in other animals.

Although several genes of the Hpo pathway and their genetic interactions are evolutionarily conserved in *C. elegans*, they are not involved in apoptosis or proliferation of cells, but is involved in cell polarity, which may reflect a more ancient function of the Hpo pathway. Similarly, in this study, we have shown for the first time that YAP-1, the main downstream effector of the Hpo pathway, is also involved in the asymmetric development of specific neurons. Our results suggest that YAP may play an important role in the neuronal polarization in other animals as well. Finally, we propose that *C. elegans* can be used as a disease model for neuronal abnormalities, based on the traits seen in *yap-1* mutant animals.
